# Bilateral symmetry of breast tissue composition by magnetic resonance in young women and adults

**DOI:** 10.1007/s10552-014-0351-0

**Published:** 2014-01-30

**Authors:** S. Hennessey, E. Huszti, A. Gunasekura, A. Salleh, L. Martin, S. Minkin, S. Chavez, N. F. Boyd

**Affiliations:** 1 Campbell Family Institute for Breast Cancer Research, Ontario Cancer Institute, 10-415 610 University Ave., Toronto, ON Canada; 2 Research Imaging Centre, Centre for Addiction and Mental Health, Toronto, ON M4T 1C8 Canada

**Keywords:** Breast cancer risk factor, Breast tissue composition, Magnetic resonance measurements

## Abstract

**Background:**

Some reports suggest that there is a slightly higher frequency of breast cancer in the left breast compared with the right in middle-aged women. The reasons for this association are unknown. The water and fat content of both breasts was compared using magnetic resonance (MR). Breast water by MR reflects fibro-glandular tissue and is strongly positively correlated with percent mammographic density, a strong risk factor for breast cancer.

**Methods:**

Magnetic resonance was used to measure fat and water content of the breast in 400 young women aged 15–30 years and a random sample of 100 of their mothers. All MR examinations were carried out using a 1.5T MR system, and 45 contiguous slices were obtained in the sagittal plane. One reader identified the breast tissue in the image, and subsequently, fat and water content was calculated using a three-point Dixon technique. Left- and right-sided images were read independently in random order.

**Results:**

In young women, mean percent water was on average 0.84 % higher in the right compared with the left breast (*p* < 0.001) and total breast water was on average 6.42 cm^3^ greater on the right side (*p* < 0.001). In mothers, there were no significant differences in any breast measure between right and left sides.

**Conclusion:**

The small differences in breast tissue composition in young women are unlikely to be associated with large differences in breast cancer risk between sides. The reported excess of left-sided breast cancer in older women is unlikely to be explained by differences in breast tissue composition.

## Introduction

Some reports suggest that there is a slightly higher frequency of breast cancer in the left breast compared with the right, with reported ratios between 1.04 and 1.26 [1–18]. Two of the largest studies, one from Sweden and the other from the USA, both showed left-sided predominance of breast cancer in Caucasian populations [13, 14]. However, not all studies show a left-sided predominance of breast cancer [12, 19–23]. Attempts to explain the left-sided predominance by examining risk factors for breast cancer, including ethnicity [8, 16], morphological type [9], race [14, 16], age [8, 9, 13–16, 24], height and weight [8], age at menarche [8, 13], family history [8], handedness [8, 13, 23–26], reproductive history [15], lactation history [19], marital status [8, 14], estrogen receptor status [8, 14, 15], parous status [8, 12, 13, 15], menopausal status [8, 13], and breast size [8, 12], have failed to consistently account for the higher incidence of left-sided breast cancer.

Breast density as measured by X-ray mammography is a strong, quantitative risk factor for breast cancer. Breast density reflects fibro-glandular tissue, comprised of epithelial and stromal tissue in the breast. The association between breast density and breast cancer is stronger than for most other breast cancer risk factors, with the exception of a few genetic factors and age [27]. Women are four to six times more likely to develop breast cancer if their breasts are composed of 75 % of more dense tissue when compared with women with little or no dense tissue. Attributable risk estimates suggest that breast density may account for as many as 30 % of breast cancer patients [28]. Previous studies in middle-aged women comparing mammographic density between left and right breasts have shown a strong bilateral symmetry [29–32]. To the best of our knowledge, mammographic density has not been examined in relation to the laterality of breast cancer.

Percent breast water as measured by magnetic resonance (MR) is strongly correlated with percent breast density, as measured by X-ray mammography [31, 33]. In the present study, we have used magnetic resonance to compare the water and fat content of both breasts in young women aged 15–30 years with a sample of their mothers.

## Methods

The present paper is based on previous work in which young women and their mothers were recruited to a study of breast tissue composition and other risk factors for breast cancer. The methods used have been published elsewhere and will be described only briefly here [34].

### Study population: selection and recruitment

Young women aged 15–30 years and their mothers were recruited from Toronto schools and clinics between December 2003 and December 2007. All daughters were healthy Caucasian young women and were excluded if they did not have regular menstrual cycles with <5 days of variation in cycle length. Also excluded were women with breast implants, augmentation or reduction breast surgery, and previous breast cancer.

Young women were required to participate in an in-person interview to complete an epidemiological questionnaire. The questions focused on demographic, menstrual, and reproductive characteristics. Height and weight were measured. Mothers completed a questionnaire that included questions about their daughters’ early life.

### Magnetic resonance imaging

All of the participating daughters and a random selection of 100 mothers had a breast MR scan. All MR examinations were carried out with an MR breast coil (Medical Advances, Milwaukee, WI, USA) using a 1.5T Signa Cvi MR system (GE, Waukesha, WI, USA). A series of 45 contiguous slices were obtained in the sagittal plane. The images were acquired with a high spatial resolution to optimize fat and water content measurements based on a three-point Dixon technique [35]. The images captured slices 7 mm thick using a 28-cm field of view with an acquisition matrix 256 × 128. The in-plane resolution was 28 cm/256–1.1 mm. A modified fast spin-echo (FSE) sequence was used with Dixon echo shifts of 0, π, and 2π and with TE/TR = 18 ms/2,500 ms. The total imaging time was 13 min. A sequence of custom-built phantoms was used to acquire the series of images calibrated for water, fat, and volume percentages. The phantoms consisted of two sizes of polypropylene spherical shells (diameter 10 and 7 cm) filled with water/oil emulsions (20 and 60 % oil). During the study, there was consistent verification of stability and accuracy by implementing a quality-control program for all MR measurements. This calibration was executed bimonthly using the identical MR procedure to verify volume precision within 2 % and water and oil content within 3 %. All MR studies took place within 10 days of the beginning of the subjects’ most recent menstrual period.

### Breast tissue measurements

#### Inter-reader reliability

A subset of the breast measurements obtained by two separate readers (reader 1: SH and reader 2: NFB) were used to assess the inter-observer reliability. Reader 1 had little measurement experience, whereas reader 2 is experienced. Both readers measured fourteen breast images.

#### Order of measured images

Breast water measurements of the left and right breasts were all completed by the same reader (SH). Measurement bias was avoided by ensuring that measurements made on one breast did not influence those of the opposite breast. The reader was “blind” to the side of the breast that was being measured, and left and right sides were presented in random order and measured on different days.

#### Measurement of images

Breast MR images were imported into a locally developed, semi-automated image analysis program for measurement. Breast tissue segmentation was achieved by using a snaking algorithm which made the chest wall and anterior breast delineation operator independent (although there was the ability to correct the line placement if need be). The only operator-dependent delineation of the breast region was in the superior and inferior cutoff regions. The three-point Dixon method was used for water/fat separation [36]. This method exploits the known chemical shift of 3.5 ppm between water and fat MR signals. It uses three complex MR signals, acquired with fat and water signals at 0, π, and 2π phase offsets, to determine the contribution of water and fat signals contained within each voxel. The software automatically calculated the water and fat content within each voxel and subsequently summed the fat and water measurements over all breast voxels within a slice and then over all slices. The measurements are shown as percent water, and the total breast volume and volumes of fat and water in cubic centimeters.

### Statistical analysis

To evaluate the inter-reader reliability and intra-reader reliability, we computed Pearson’s correlation coefficients and used simple linear regression models.

All breast measurements in daughters and mothers showed a skewed distribution and were transformed to make the distributions more symmetrical with stable variance. Percent water measurements were log-transformed, and volume measurements (total fat, total water, and total volume) were cubic-root-transformed. To aid interpretation, untransformed breast tissue measurements are shown in box-plots around the median values. Bland–Altman plots [37], *with untransformed values* of the percent relative differences (in percent water) of the right breast relative to the left, are also shown.

Subsequently, the transformed values were used to calculate the absolute differences in breast tissue between sides. We calculated means and standard deviations and ascertained the differences between left and right breasts by using a paired *t* test on the transformed scale. A positive difference indicates a larger left breast measurement. A p value of < 0.05 was used to determine the statistical significance. Absolute (positive) differences in breast tissue measurements between left and right breasts for both daughters and mothers were also calculated. Since the distributions of absolute differences were skewed, medians and corresponding 95 % confidence intervals are shown [38]. Statistical analysis was carried out using Minitab 12.22 and S-plus 6.2.

## Results

### Reliability of measurements

#### Inter-reader reliability

**Fig. 1:**
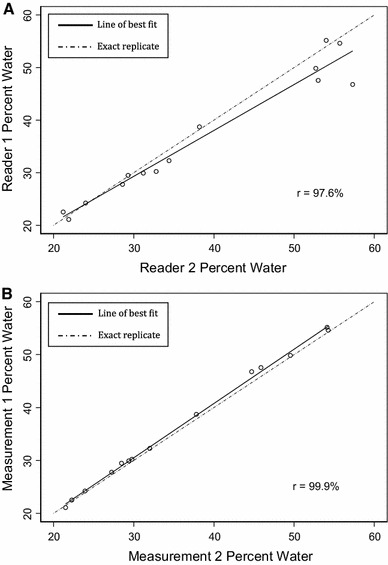
**a** Inter-reader reliability of measurements. **b** Intra-reader reliability of measurements. *Lines* were obtained using univariable linear regression

Figure 1a compares measurements of breast images obtained from the two investigators for percent water. Values obtained by reader 1 are slightly but systematically lower than those obtained by reader 2, with somewhat larger differences at higher values. However, for percent water, correlation was 97.6 %. Similar correlations, ranging from 97.3 to 97.9 %, were obtained for total fat, total water, and total volume (data not shown), indicating close associations with the values measured by both readers.

#### Intra-reader reliability

To further assess the reliability of the methods, reader 1 measured the same sample of fourteen breast images on two occasions (measurement 1 and measurement 2). A comparison between these percent water measurements is shown in Fig. 1b and has a correlation of 99.9 %. The plot shows that there are no systematic differences between the two reads. Similar correlations, ranging from 99.4 to 99.8 %, were obtained for total fat, total water, and total volume, indicating a close association between readings (data not shown).

### Characteristics of subjects

Table 1 shows selected data obtained by questionnaire from mothers (*n* = 100) and daughters (*n* = 400). The average age was 49.6 years for mothers and 20.8 years for daughters. The average weight of mothers was 64.7 kg and of daughters 60.6 kg. Average height was 164.2 cm for mothers and 165.8 cm for daughters. Thirty percent of mothers were postmenopausal, and four percent were currently using hormone replacement therapy. Thirty percent of daughters used oral contraceptives and 6.5 % were parous.

**Table 1:** Characteristics of subjects

	Mothers (*n* = 100)	Daughters (*n* = 400)
Age at time of study (years)	49.6 (4.2)	20.8 (4.9)
Weight (kg)	64.7 (10.3)	60.6 (10.5)
Height (cm)	164.2 (6.6)	165.8 (5.9)
Body mass index (kg/m^2^)	24.0 (3.7)	22.0 (3.4)
Age at menarche (years)	12.8 (1.4)	12.5 (1.3)
Current alcohol consumption at least 1 × week (%yes)	63.6	24.8
Current smoke intake at least 1 cigarette/day (% yes)	5.1	7.0
Current hormone replacement use (% yes)	4.0	0
Current oral contraceptive use (% yes)	6.1	30.0
Menopausal status (% postmenopausal)	30.3	0
Pregnant ever (% yes)	100	6.5
No first-degree relatives with breast cancer (%)	71.0	92.2
1 first-degree relative with breast cancer (%)	27.0	7.5

Characteristics are given as mean (SD)

### Daughters’ breast tissue measurements

The distributions of breast measurements for percent water, total fat, total water, and total volume were all positively skewed. After log transformation of the percent water measurements and cubic root transformation of the volume measurements, the data were approximately symmetrically distributed (data not shown).

**Fig. 2:**
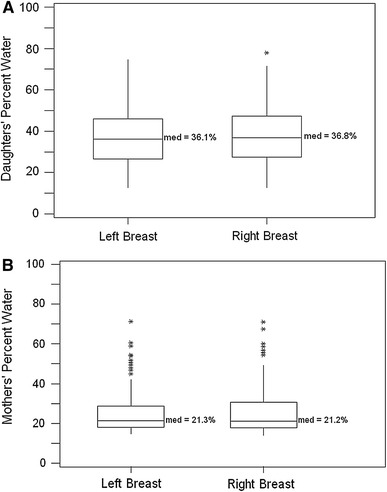
*Box-plots* comparing the **a** daughters’ (*n* = 400) and **b** mothers’ (*n* = 100) percent water content of the *left* breast versus *right* breast

The untransformed distribution of values for percent water is shown in box-plots in Fig. 2a. Compared with the left breast, the right breast had a 0.7 % higher median for percent water.

Table 2 shows breast tissue measurements in the left and right breasts of daughters, with means back-transformed to the original scale. The average percent water was 0.84 % (*p* < 0.001) higher in the right compared with the left breast in daughters. The median absolute difference in percent water between left and right breasts was 1.61 % (95 % CI 1.40; 1.86). The average total breast water volume was 6.42 cm^3^ (*p* < 0.001) greater on the right side in daughters with a median absolute difference of 16.3 cm^3^ (95 % CI 14.0; 17.6). The difference between left and right breasts was 2.63 and 5.30 cm^3^ in mean total fat and total volume, respectively, none of them statistically significant.

**Table 2:** Comparison of daughters’ left breast composition with right breast composition as measured from MR images (*n* = 400)

	Daughters’ MRI breast measurements			
	Percent water	Total fat (cm^3^)	Total water (cm^3^)	Total volume (cm^3^)
Mean^a^ (95 % CI)				
Left	34.64 (33.4; 35.87)	371.08 (350.40; 392.22)	204.75 (196.12; 213.85)	591.22 (565.61; 618.47)
Right	35.48 (34.12; 36.97)	368.45 (347.43; 390.62)	211.18 (202.26; 220.35)	596.52 (569.72; 622.84)
Difference between means^b^ (*p* value)	−0.84 (<0.001)	2.63 (0.23)	−6.42 (<0.001)	−5.30 (0.129)
Median absolute difference (95 % CI)	1.61 (1.40; 1.86)	23.5 (21.7; 26.0)	16.3 (14.0; 17.6)	35.3 (29.8; 39.9)

^a^Back-transformed means

^b^Untransformed values

**Fig. 3:**
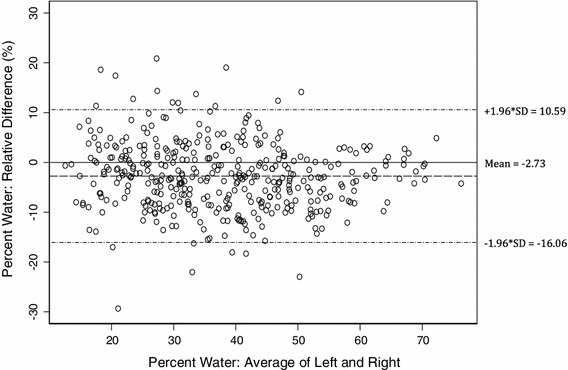
Bland–Altman plot displaying the relative difference in percent water between the *left* and *right* breasts for daughters using untransformed values (*n* = 400)

The Bland–Altman plot for young women (Fig. 3) displays the percentage of relative difference in percent water between the left and right breasts versus the average percent water of both breasts using untransformed values. It shows that for breasts of average density, the right breast will have on average 2.73 % greater percent water compared with the left. The plot also shows a slightly higher variation in the relative difference between the two breasts at lower values of average percent water.

### Mothers’ breast tissue measurements

The distributions of breast measurements for percent water, total fat, total water, and total volume were all positively skewed. The data became approximately symmetrically distributed after log transformation of percent water values and cubic root transformation of volume measurements (data not shown).

The untransformed distribution of values for percent water is shown in box-plots in Fig. 2b. In mothers, the left breast had a 0.1 % higher median percent water compared with the right breast. Median percent water was 21 % in mothers (Fig. 2b) compared with 36 % in daughters (Fig. 2a).

The median absolute difference was 0.97 % (95 % CI 0.80; 1.21) in mothers. The back-transformed average percent water was 0.17 % (*p* = 0.32) higher in the right breast compared with the left. None of the differences between the right and left breasts in mean total fat, total water, and total volume in mothers were statistically significant (Table 3).

**Table 3:** Comparison of mothers’ left breast composition with right breast composition as measured from MR images (*n* = 100)

	Mothers’ MRI breast measurements			
	Percent water	Total fat (cm^3^)	Total water (cm^3^)	Total volume (cm^3^)
Mean^a^ (95 % CI)				
Left	23.95 (22.20; 25.79)	545.54 (481.89; 614.13)	172.99 (159.22; 187.15)	733.14 (663.05; 809.56)
Right	24.12 (22.20; 26.05)	551.97 (483.74; 625.03)	175.71 (161.88; 190.11)	743.68 (667.63; 825.29)
Difference between means (*p* value)	−0.17 (0.32)	−6.43 (0.33)	−2.72 (0.31)	−10.54 (0.25)
Median absolute difference^b^ (95 % CI)	0.97 (0.80; 1.21)	34.3 (26.1; 56.6)	14.0 (10.5; 19.6)	48.7 (37.8; 73.8)

^a^Back-transformed means

^b^Untransformed values

**Fig. 4:**
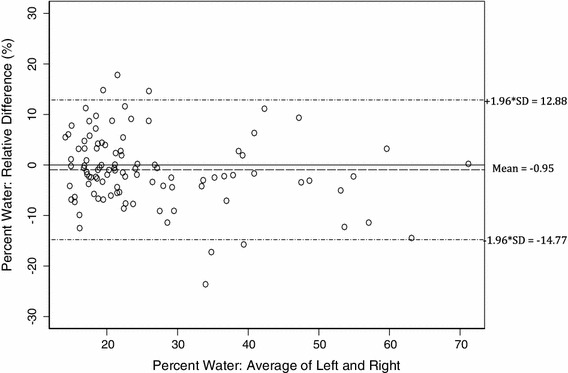
Bland–Altman plot displaying the relative difference in percent water between the *left* and *right* breasts for mothers using untransformed values (*n* = 100)

The Bland–Altman plot (Fig. 4) shows the percent water in the right breast of mothers to be higher by 0.95 % on average compared to the left breast, in relation to breasts of average density using untransformed values. No systematic pattern was observed in the variation in the relative differences between breasts along the axis of the average values of percent water.

## Discussion

The factors that influence the laterality of breast cancer risk are currently unknown. Mammographic density is a strong, heritable risk factor for breast cancer that, to the best of our knowledge, has not previously been examined in relation to breast cancer laterality. Percent water content as measured by MR is strongly correlated with percent breast density as measured by X-ray mammography [31], and they both reflect fibro-glandular tissue in the breast (the small amount of water in fat is taken into account and is not included in the calculation of percent water).

In this study, we compared breast water content between the left and right breasts in young women, and our results show that percent water and total water differ slightly between breasts, with the right breast having small but statistically significant higher average values. In previous work, in adult women, we have estimated that a 1 % difference in percent mammographic density is associated with a 2 % difference in the relative risk of breast cancer [39]. If a similar relationship between breast tissue composition and breast cancer risk exists in young women, the small observed median absolute difference in percent water is unlikely to be associated with large differences in breast cancer risk between sides, such as was seen in a large, population-based Swedish study [13]. That study showed a higher risk of right-sided breast cancer (left–right risk ratio of 0.87; 95 % CI 0.75; 1.00) in a subset of nulliparous women under the age of 45, which differed significantly from the left–right risk ratio at all ages of 1.02.

In contrast to our results in young women, we observed no significant differences in tissue composition between the left and right breasts in middle-aged, parous women. This is consistent with previous studies comparing mammographic density between left and right breasts in middle-aged women that have shown strong bilateral symmetry, suggesting that breast cancer risk can be accurately predicted using measurement of only one breast [29–32]. Furthermore, the similarities between sides seen in middle-aged women suggest that any difference in the laterality of breast cancer in middle age and later is not likely to be explained by differences in breast density.

Strengths of this study include the method of breast tissue measurement that is volumetric and quantitative, requires minimal human involvement, and is highly reliable. Both the inter-reader and intra-reader reliability results show a strong positive correlation between the breast tissue measurements taken at different times and by different readers. Further, all measurements were taken by one individual (SH), of known reliability, and we ensured that measurements of the left and right breasts in each individual were taken independently of each other.

We have, however, examined only the bilateral symmetry of breast tissue composition, a known risk factor for breast cancer in midlife and later, and have not directly assessed breast cancer risk. It remains possible that variations in the epithelial or stromal components of fibro-glandular tissue, which cannot be distinguished by the MR measurement of water used here, may show asymmetry that is not reflected in the overall measure of fibro-glandular tissue.
